# Challenging the safety and efficacy of topically applied chlorogenic acid, apigenin, kaempferol, and naringenin by HET-CAM, HPLC-TBARS-EVSC, and laser Doppler flowmetry

**DOI:** 10.3389/fchem.2024.1400881

**Published:** 2024-05-20

**Authors:** Nadia Ruscinc, Ricardo Augusto Massarico Serafim, Cíntia Almeida, Catarina Rosado, André Rolim Baby

**Affiliations:** ^1^ Department of Pharmacy, Faculty of Pharmaceutical Sciences, University of São Paulo, São Paulo, Brazil; ^2^ Department of Pharmaceutical/Medicinal Chemistry, Eberhard Karls University Tubingen, Tubingen, Germany; ^3^ CBIOS-Universidade Lusófona’s Research Center for Biosciences and Health Technologies, Lisbon, Portugal; ^4^ Department of Biomedical Sciences, University of Alcalá, Madrid, Spain

**Keywords:** polyphenols, cosmetics, stratum corneum, anti-inflammatory, antioxidant

## Abstract

The integumentary system, a vital organ, constitutes a multifaceted barrier against pathogens and environmental factors, crucial for maintaining homeostasis. Intrinsic and extrinsic factors can accelerate skin aging and compromise its homeostatic functions and solar rays, particularly ultraviolet (UV) radiation, pose a significant risk for skin cancer. Polyphenols are molecules that donate hydrogen or electrons, preventing the oxidation of substances, such as lipids, or the formation of inflammatory mediators by cyclooxygenase enzymes. This study explored the *in vitro* safety, by HET-CAM (hen’s egg test on chorioallantoic membrane), and protective effects of polyphenols (chlorogenic acid, apigenin, kaempferol, and naringenin) against stratum corneum UV-induced lipid peroxidation using an innovative method, the HPLC-TBARS-EVSC (high-performance liquid chromatography–thiobarbituric acid reactive substances–*ex vivo* stratum corneum), and a stress test using methyl nicotinate and laser Doppler flowmetry to establish *in vivo* the samples’ topical anti-inflammatory ability. An aqueous gel containing 0.1% *w*/*w* of each polyphenol was formulated using ammonium acryloyldimethyltaurate/VP copolymer. Through the utilization of the HET-CAM assay for *in vitro* safety assessment, chlorogenic acid, apigenin, kaempferol, and naringenin were classified as non-irritating active ingredients. This classification was based on their lack of adverse reactions within the vascularization of the chorioallantoic membrane. To assess the protective capabilities of four polyphenols against lipid peroxidation in the stratum corneum, the HPLC-TBARS-EVSC protocol was conducted. It was observed that only naringenin exhibited a significant reduction in epidermal lipoperoxidation, indicating superior anti-radical potential. Conversely, chlorogenic acid, apigenin, and kaempferol displayed a pro-oxidant profile under the specified test conditions. The laser Doppler flowmetry suggested the anti-inflammatory potential of naringenin, kaempferol, and chlorogenic acid, with naringenin showing superior efficacy involving all parameters quantified. Naringenin emerged as the only polyphenol capable of reducing the intensity of the inflammatory response induced by methyl nicotinate solution in the participants, compared to the blank gel and the untreated area. This comprehensive investigation underscores the diverse protective roles of polyphenols in skin health, emphasizing naringenin’s notable anti-radical and anti-inflammatory properties.

## 1 Introduction

The integumentary system is an organ system with a specialized structure that forms a protective barrier around the external aspect of an organism. This barrier defends against pathogens and environmental factors, regulates water and electrolyte balance, synthesizes vitamin D, and helps regulate body temperature to maintain homeostasis (Junqueira and Carneiro, 2013). Comprising the epidermis, dermis, and hypodermis, the skin has a tri-layered architecture, with each layer having distinct characteristics and functions (Kolarsick et al., 2011). Maintaining these layers’ structural integrity and functionality is crucial due to the skin’s vital role in preserving life.

The skin is subject to intrinsic and extrinsic processes that can accelerate cellular senescence, disrupting its homeostatic functions (Tobin, 2017). A proactive approach to sustaining skin health involves the use of products that protect the tissue against solar rays, especially ultraviolet (UV) radiation, as unprotected exposure poses a significant risk for skin cancer development (IARC, 2012) and other degenerative processes, like actinic elastosis (Reinehr, 2019; Sinová et al., 2021). Skin cancer, a prevalent malignancy in the Brazilian population, causes substantial harm, characterized by the abnormal proliferation of skin cells (Zhao et al., 2017). Projections from the National Cancer Institute (INCA) anticipate 220,490 new cases of non-melanoma skin cancer annually between 2023 and 2025, corresponding to 101.95 new cases per 100,000 inhabitants (INCA, 2022). Despite endogenous defense mechanisms, cumulative exposure to harmful UV effects can disrupt skin homeostasis, predisposing the skin to pathologies and accelerating aging processes. Natural protective mechanisms, such as the inhibition of free radicals and thickening of the horny layer, have limits in the face of prolonged exposure (Jansen et al., 2013).

**FIGURE 1:**
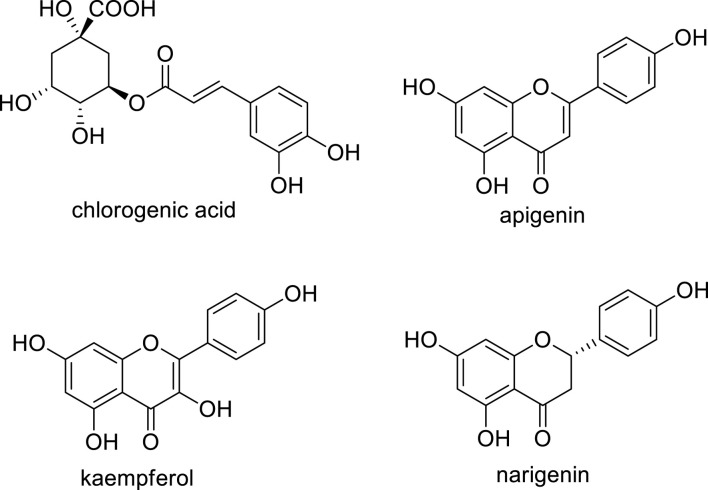
Chemical structures of the polyphenols evaluated in this investigation.

Polyphenols are molecules that readily donate hydrogen or electrons, effectively preventing the oxidation of substances such as lipids (Velloza et al., 2021), or the formation of inflammatory mediators by cyclooxygenase (COX) enzymes (Sauce et al., 2021). These properties allow them to inhibit the formation of reactive oxygen species (ROS) and mitigate the harmful effects caused by UV radiation (Sun et al., 2022). This protective mechanism of action against various adverse contexts justifies the present investigation, in which we established the *in vitro* safety of the topical application of chlorogenic acid, apigenin, kaempferol, and naringenin (chemical structures illustrated in Figure 1). We also innovated by using the *ex vivo* assay developed by our research group, the HPLC-TBARS-EVSC (high-performance liquid chromatography-thiobarbituric acid reactive substances-*ex vivo* stratum corneum) (Sauce et al., 2021), and determined *in vivo* the anti-inflammatory potential of these polyphenols through a stress test using methyl nicotinate and laser Doppler flowmetry.

## 2 Experimental

### 2.1 Samples

An aqueous gel containing chlorogenic acid, apigenin, kaempferol, or naringenin was formulated using the ammonium acryloyldimethyltaurate/VP copolymer (Aristoflex AVC), an emulsifying polymer (Ariede et al., 2020). The active ingredient was added incrementally until a clear and transparent gel was achieved, with a pH value of 6.5. Table 1 outlines the starting materials and their respective proportions (% w/w) used in this study.

**TABLE 1:** Qualitative and quantitative composition (% *w*/*w*) of the samples.

Ingredients	Concentration (% *w*/*w*)				
	*API*	*KAPF*	*ACLOR*	*NAR*	*ARX*
Ammonium acryloyldimethyltaurate/VP copolymer	0.3	0.3	0.3	0.3	0.3
Apigenin	0.1	-	-	-	-
Kaempferol	-	0.1	-	-	-
Chlorogenic acid	-	-	0.1	-	-
Naringenin	-	-	-	0.1	
Aqua (purified water)	99.6	99.6	99.6	99.6	99.7

### 2.2 *In vitro* safety establishment by the HET-CAM methods

The HET-CAM (hen’s egg test on chorioallantoic membrane) assay involved the use of fertilized white Leghorn chicken eggs, which were incubated for 10 days at 37°C and 65% relative humidity in a Chocmaster incubator. After incubation, the shell around the air chamber was delicately removed with tweezers, exposing the shell membrane. The membrane was then hydrated with 300 μL of 0.9% w/v NaCl solution (FS) and subsequently removed. The chorioallantoic membrane of each egg was exposed, and 300 μL of a solution containing the samples dispersed in FS was applied to it. The positive control consisted of a 1.0% *w*/*v* solution of sodium dodecyl sulfate (SDS), while the negative control was the FS. Each egg containing the sample was placed in a fixed support, and a camera was positioned to capture the chorioallantoic membrane with clarity, highlighting blood vessels against the yellow yolk or vitelline sac in the background. Vascular events, such as bleeding, lysis, or coagulation, were recorded for 5 minutes using a Dino-Lite camera (Model AM-211). Subsequently, the events were assessed and analyzed. The irritation index (IS) was calculated using Equation 1 (Kalweit et al., 1990; National Institute of Environmental Health Sciences, 2021; Ruscinc et al., 2022). All assays were conducted in triplicate, and the samples were classified according to Table 2.

$$\text{IS}=\left(301-H300\right)\times 5+\left(301-L300\right)\times 7+\left(301-C300\right)\times 9IS$$

$$=\left(300301-H\right)\times 5+\left(300301-L\right)\times 7+\left(300301-C\right)\times 9$$

**TABLE 2:** Classification of irritation potential (irritation index) by the HET-CAM test (Kalweit et al., 1990).

Irritation index	Classification
0–0.9	Non-irritating
1.0–4.9	Mildly irritating
5.0–8.9	Moderately irritating
9.0–21	Strongly irritating

Eq 1. Calculation of the irritation index (IS). H–time (s) to initiate a hemorrhagic event; L–time (s) to initiate vascular lysis; C–time (s) to initiate coagulation.

### 2.3 *Ex vivo* and *in vivo* efficacy evaluation

Participants in this investigation were provided with comprehensive information and clarification regarding the study’s purpose and methodology. They demonstrated the capacity to understand and adhere to the test procedures. Consent was obtained through the signing of a consent form, ensuring participant anonymity and the option for voluntary withdrawal at any stage (Marques et al., 2023). The project received approval from the Ethics Committees of the Faculty of Pharmaceutical Sciences, University of São Paulo (protocol 4.885.215; CAAE49880121.0.0000.0067), and Lusófona University (protocol CE. ECTS/P11.21). The studies were conducted following current legislation, ethical principles, norms on human experimentation, and the Declaration of Helsinki. Inclusion criteria stipulated that participants must be non-smokers, have healthy skin without any reported dermatological or circulatory pathology, be aged between 18 and 60 years, and have Fitzpatrick skin types II-V. Exclusion criteria included individuals with active atopic dermatitis, intense sun exposure up to 4 weeks before the study, allergy to the product category used, pregnant or breastfeeding women, and use of immunosuppressive medications, corticosteroids, antihistamines, retinoids, and anti-inflammatories. Participants were advised not to apply other cosmetic products to the assessment area 24 h before the experiment. The volunteers rested for, at least, 15 min under a controlled temperature of 21°C ± 2°C and a relative humidity of 40%–60% before any measurements were made.

#### 2.3.1 HPLC-TBARS-EVSC (high-performance liquid chromatography–thiobarbituric acid reactive substances–*ex vivo* stratum corneum) protocol

The volar forearm of eight participants underwent a single cleaning using dry cotton. Subsequently, aliquots of the samples weighing, approximately, 19.0 mg (delimited area of 9.5 cm^2^; ratio of 2.0 mg/cm^2^), containing each of the evaluated polyphenols (Table 1), and the blank gel, were applied to the pre-defined areas. After 2 h of application, the stratum corneum from the site was removed using the tape-stripping technique (Marques et al., 2023).

##### 2.3.1.1 Tape stripping procedure

The tape stripping method was used for *ex vivo* obtaining the stratum corneum (SC) from the participants. Delimited areas on the volar forearm of volunteers were treated with or without the samples as described earlier. The samples and the control were applied using a disposable plastic spatula, ensuring even distribution over the defined area. The application proportion was determined based on current recommendations for sunscreens. After 2 h of application, the tape stripping technique was employed using 6 strips per area to remove the SC (Alonso et al., 2009). The application of samples, control, and untreated area was randomized. Strips were applied with consistent pressure at each designated location for SC removal. The first strip from each site was eliminated, and the subsequent 5 strips were processed (Sauce et al., 2021). The removed strips were irradiated (exposed to UV radiation) using a photostability chamber (Atlas Suntest CPS+) equipped with a xenon lamp (1500 W) and a filter that simulates solar radiation by allowing wavelengths above 290 nm to pass through. Irradiation was conducted at 765 W/m^2^, with a fixed dose and a determined period of 2 h. The temperature was controlled at 35°C to prevent sample overheating (Marques et al., 2023).

##### 2.3.1.2 Extraction of the stratum corneum

All collected adhesive strips were transferred to previously labeled 50.0 mL Falcon tubes. Subsequently, all samples in the tubes were treated with 10.0 mL aliquots of HPLC-grade methanol. The extraction of the SC by the solvent was carried out using an ultrasonic bath for 15 min. The extracted stratum corneum was evaluated using the HPLC-TBARS-EVSC protocol (Marques et al., 2023).

##### 2.3.1.3 Quantification of the lipid peroxides of the stratum corneum

Lipid peroxides from the tape-stripped SC were quantified using the HPLC, following the method described by Marques and colleagues, 2023. The chromatography was performed isocratically with a mobile phase composed of 35% methanol and 65% phosphate buffer, *v*/*v* (50 mM, pH 7.0). The flow rate was set at 1.0 mL per minute for 10 min at a temperature of 30°C, with a sample injection volume of 40.0 µL. A diode detector was set at 532 nm to quantify the MDA-TBA_2_ (MDA–malondialdehyde; TBA–2-thiobarbituric acid) adduct (Bastos et al., 2012; Sauce et al., 2021). Using an automatic pipette, 2.0 mL of the samples of the extracted SC were transferred to glass tubes with lids and identified accordingly. Subsequently, 800.0 µL of H_3_PO_4_ (0.44M) and 288.0 µL of BHT (butylated hydroxytoluene) (0.2% in methanol) were added to the tubes and vortexed for 1 minute. The mixture was then allowed to stand for 10 min at room temperature in the dark. Next, 1,200.0 µL of TBA (0.6% in H_3_PO_4_ - 0.44M) were added, and the tubes were vortexed for 30 s. The tubes were placed in a thermostatic bath and heated to 90°C, remaining for 45 min. After cooling to room temperature, 1,200.0 µL of n-butanol were added to the tubes, and vortexed for 1 minute. Subsequently, an aliquot of the tube contents was filtered through a 0.22 µm filter attached to a syringe with a needle, directly into the identified vial for the determination of the lipid peroxidation profile of the SC concerning the treatment, by quantifying the MDA-TBA_2_ adduct (Bastos et al., 2012; Pescia et al., 2012; Frantzen et al., 2016; Marques et al., 2023). The results were evaluated by the ratio of the irradiated sample to the non-irradiated SC.

#### 2.3.2 *In vivo* anti-inflammatory activity assay

Six areas were marked on the mid-volar forearm of 14 participants. Five areas were designated for sample application, while one served as the untreated control. The experiment was conducted in a temperature-controlled environment (21°C ± 2°C and relative humidity of 40%–60%). Before treatment, the designated areas were gently cleaned with a cotton disc, and 2.0 mg/cm^2^ of the sample was applied using a spatula. The samples were randomly assigned to either the left or right forearm. After 2 h, a vasodilatory response was induced in each area by applying for 60 s a filter paper with 1.0 cm^2^ saturated with 0.5% (*w*/*v*) methyl nicotinate aqueous solution. Immediately after removing the filter paper and wiping any remaining liquid, blood flow measurements were continuously made for 15 min at each test site using a Laser Doppler Flowmetry system (PeriFlux System 5,000, Perimed). The onset time (t_onset_), area under the curve, and angular coefficient were obtained using the Laser Doppler flowmetry equipment and analyzed through PeriSoft software, version 2.5.5. To decrease the impact of the inter and intra-variability, results were analyzed based on the ratio of the values obtained at each sample site to the control values for each participant (Cândido et al., 2022).

### 2.4 Statistical analysis

Statistical evaluation was conducted using one-way ANOVA and non-parametric statistics (Wilcoxon test) with a significance level of 0.05. GraphPad Prism version 5.0 software (GraphPad Software, Inc.) was employed for the analysis.

## 3 Results and discussion

The HET-CAM assay is an alternative method to animal experimentation, based on the similarity between the chorioallantoic membrane of a developing chicken egg and the mucosa of the human or rabbit eye, due to the similar vascularized tissue (Kalweit et al., 1990; Steiling et al., 1999). Establishing the *in vitro* safety profile of polyphenols, particularly, chlorogenic acid, apigenin, kaempferol, and naringenin, was significant as a preliminary assay for the future application of active ingredients in a suitable semi-solid vehicle or, considering the market, a finished cosmetic product, in other words, preserving the safety of the participants and consumers. It is worth noting that products applied to the facial area may encounter the ocular region, thus expanding the rationale for the HET-CAM test. The *in vitro* method was chosen due to its convenient conduct, availability, and generation of results comparable to the Draize Test, which is performed on rabbits and is currently not acceptable for testing starting materials or cosmetic products (ALLTOX, 2016). Furthermore, the assay is less costly, requires less complex infrastructure, and does not involve the participation of subjects (Wang et al., 2011).

Interagency Coordinating Committee on Validation of Alternative Methods, 2010, advocates that HET-CAM assay results can be accepted only if the positive control, 1.0% *w*/*v* SDS, exhibits an IS that classifies it as strongly irritating. The positive control showed an acceptable result, with hemorrhage and lysis observed within a few seconds (mean time = 2 s). The negative control FS (0.9% *w*/*v* NaCl) did not trigger vascular alteration processes, as can be seen in Table 3. Thus, considering that the controls confirmed the adequacy of the assay, the results for chlorogenic acid, apigenin, kaempferol, naringenin, and the polymer were acceptable.

**TABLE 3:** Irritation potential, by HET-CAM test, of the chlorogenic acid, apigenin, kaempferol, naringenin, and the blank gel.

Sample	At (S) hemorrhagic event	At (S) vascular lysis	IS	Classification
Chlorogenic acid	0.73	No events	0.73	NI
Apigenin	No events		0.1	NI
Blank gel			0.1	NI
Kaempferol			0.1	NI
Naringenin			0.1	NI
FS (0.9% *w*/*v* NaCl)			0.1	NI
SDS (1.0% *w*/*v* sodium dodecyl sulfate)	2.0	2.0	11.96	SI

**FIGURE 2:**
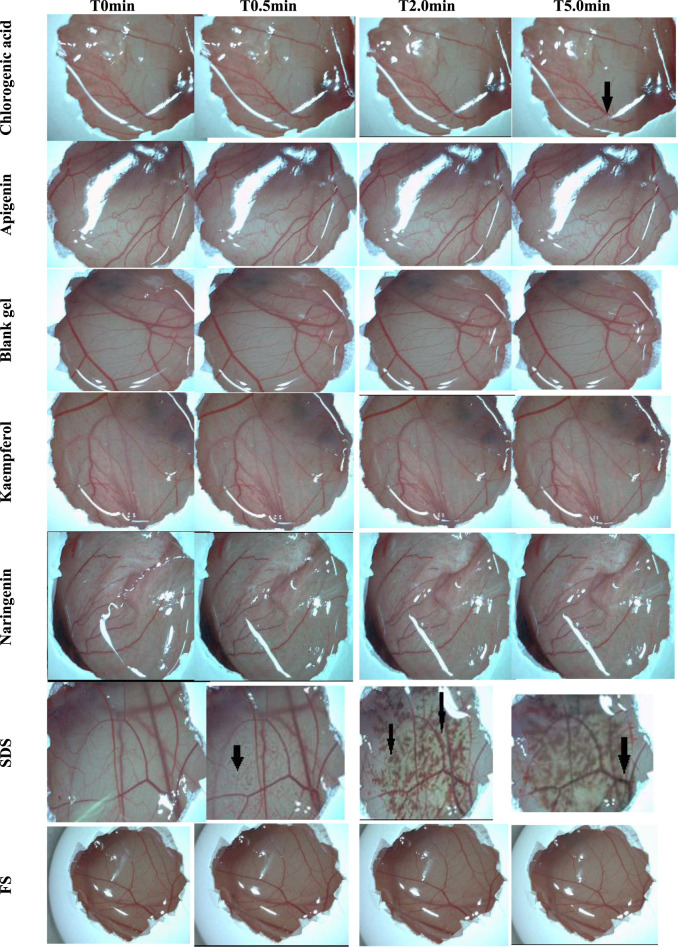
Effects of the samples and controls on the chorioallantoic membrane (image records at 0, 0.5, 2.0, and 5.0 min). The arrows point to vascular events/alterations, like hemorrhage and lysis. Legend: T0min–time 0 minutes; T0.5min–time 0.5 min; T2.0min–time 2.0 min; T5.0min–time 5.0 min; SDS–Sodium dodecyl sulfate (1.0% w/v); FS–NaCl (0.9% w/v).

The video image records at 0, 0.5, 2.0, and 5.0 min are illustrated in Figure 2. It was observed that only chlorogenic acid showed a hemorrhage event that was registered at 5 min of contact with the chorioallantoic membrane. Despite causing this event, this sample was classified as non-irritating. The other polyphenols and the polymer did not show evidence of coagulation, lysis, and/or hemorrhage in the membrane vessels; therefore, they were also classified as non-irritating. According to the results of the HET-CAM assay, all samples were suitable for further *ex vivo* and *in vivo* experiments, specifically, the HPLC-TBARS-EVSC protocol and anti-inflammatory activity assessed by laser Doppler flowmetry.

**FIGURE 3:**
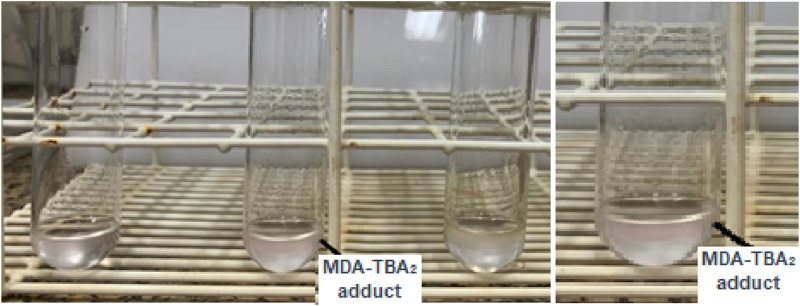
Formation of the MDA-TBA_2_ adduct through the pink supernatant formation.

The thiobarbituric acid reactive substances (TBARS) method allows the evaluation of lipid peroxides in the outer layers of the SC, formed through UV irradiation in an artificial UV simulator. This method quantifies lipid peroxidation by measuring substances reacting with TBA in the medium (Bastos et al., 2012; Sauce et al., 2021). Lipid peroxidation involves a chain reaction that affects the polyunsaturated fatty acids in cell membranes. One characteristic distinguishing these fatty acid structures is the presence of multiple double carbon-carbon bonds and reactive hydrogen atoms. Such structures are more susceptible to oxidative processes, exhibiting a direct relationship between the degree of unsaturation and the tendency to undergo oxidation (Musakhanian et al., 2022). This condition of instability facilitates the generation of free radicals, and as the cell membrane is the structure affected, it results in the process of lipid peroxidation. Consequently, significant degenerative changes are triggered, compromising cell integrity and altering permeability, fluidity, and electrical resistance, i.e., modifying the cell barrier and, consequently, weakening the intracellular environment and cell metabolism (Torres et al., 2021). These disruptions in balance promote the formation of certain components, such as MDA, a biomarker of lipid peroxidation. MDA is a product of the breakdown of polyunsaturated fatty acids and is the final product studied to assess cellular damage (Pilz et al., 2000; Del Rio et al., 2005). This component, quantified through its detection by analytical tools, indicates an increase in oxidative stress levels resulting from lipid peroxidation in a specific tissue or sample (Del Rio et al., 2005). The detection and analysis of these MDA levels in the system can be performed through the TBARS method (Yagi et al., 1994; Lima and Abdalla, 2001). This method assesses the final products of lipid peroxidation, like lipid peroxides, MDA, and various low molecular weight aldehydes. When MDA reacts with two molecules of TBA in an acidic medium, it forms the MDA-TBA_2_ adduct, a detectable fluorescent pink chromogen. Figure 3 illustrates the formation of the pink supernatant, representing the MDA-TBA_2_ adduct.

**FIGURE 4:**
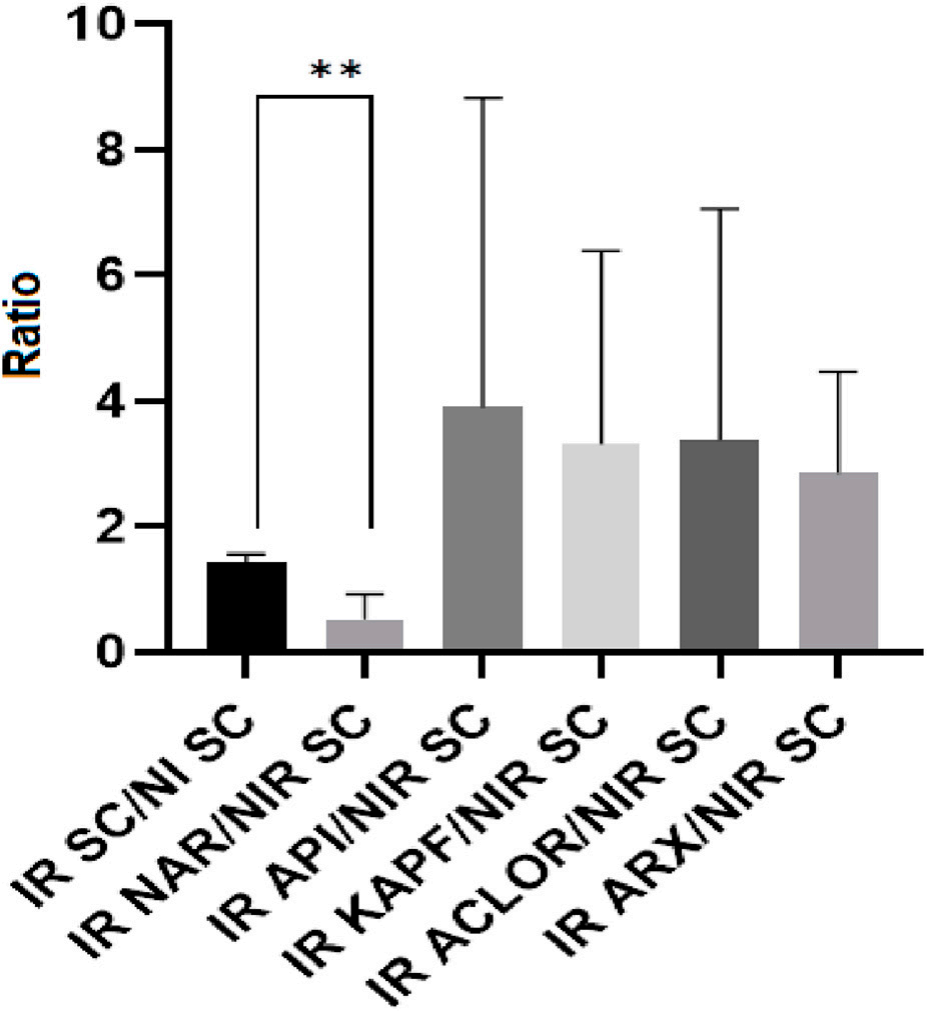
HPLC-TBARS-EVSC results for the samples as the ratio between the irradiated samples/SC and the non-irradiated (basal) SC. Legend: NI SC–non-irradiated stratum corneum; IR SC - irradiated stratum corneum; IR ACLOR - irradiated chlorogenic acid; IR API - irradiated apigenin; IR ARX–irradiated blank gel; IR KAPF - irradiated kaempferol. **p = 0.0078.

In this investigation, our developed HPLC-TBARS-EVSC protocol (Marques et al., 2023) was employed. This assay allows for improved specificity in the detection and quantification of MDA-TBA_2_, aiming to measure the profile of lipid peroxidation in the SC of participants obtained through tape-stripping (Alonso et al., 2009). The HPLC-TBARS-EVSC protocol was utilized to assess the *ex vivo* antioxidant activity of the chlorogenic acid, apigenin, kaempferol, and naringenin by evaluating their protection potential in the SC, whether exposed to artificial UV radiation. Eight volunteers participated in the assay. As illustrated in Figure 4, the determined values of the adduct in the irradiated SC were almost two-fold higher than its corresponding non-irradiated counterpart. These results show that there was a significant increase in lipid peroxidation in the sample under radiation stress conditions, validating the method.

According to the literature, the use of antioxidants enhances the body’s defense against the harmful effects of free radicals, thereby preventing the initiation and progression of lipid peroxidation (Bedard and Krause, 2007). However, treating the participants with chlorogenic acid, apigenin, and kaempferol did not provide sufficient protection for the SC against lipid peroxidation when exposed to artificial UV, as indicated by the high values in the ratios obtained from HPLC-TBARS-EVSC analysis. However, the mean ratio of the SC sample treated with naringenin (irradiated) to the non-irradiated SC (control) was around one and showed a statistically significant difference (*p* < 0.05) from the ratio of the irradiated SC to its non-irradiated counterpart, suggesting that naringenin effectively protected against the UV-induced lipid peroxidation. This finding is consistent with the results obtained by Martinez and colleagues, 2016, who observed an inhibition of superoxide radical (O2⋅–) production, and reduced levels of lipid hydroperoxides in mice skin exposed to UVB radiation and treated with a formulation containing naringenin, indicating an increase in the skin’s antioxidant capacity and suggesting potential protective activity of naringenin against UVB radiation (Kim et al., 2012; Martinez et al., 2016).

The laser Doppler flowmetry method employs a flowmeter based on optical principles to assess microvascular cutaneous perfusion (Sarnik et al., 2007). This technique allows for the indirect and continuous evaluation of changes in blood flow and the measurement of cutaneous microvascular perfusion at a specific location in contact with the probe (Petersen, 2013). The method utilizes a monochromatic laser transmitted through an optical fiber, which, upon penetrating the tissue, illuminates red blood cells in motion in the blood vessels. The laser beam undergoes a shift and is reflected at a wavelength different from the original at the time of emission, a phenomenon known as Doppler shift. This change is directly related to the speed and volume of blood cells and, thus, to vasodilation. In addition to being non-invasive, the laser Doppler flowmetry is an *in vivo* and painless technique, commonly used in studies to evaluate topical inflammatory reactions (Sikurova et al., 2011).

The stress test using methyl nicotinate and the laser Doppler flowmetry was employed to assess *in vivo* the potential topical anti-inflammatory activity of chlorogenic acid, apigenin, kaempferol, and naringenin, using the blank gel as a negative control. The parameters onset time of action (t_onset_), area under the curve (AUC), and the slope of the tangent line (angular coefficient) during the hyperemia phase were used to assess the anti-inflammatory capacity (Vertuani et al., 2003). The methodology is based on the stimulation of cutaneous microcirculation (vasodilation) by applying methyl nicotinate solution to each pre-treated site, assessing the samples under analysis for their ability to mitigate this response (Zhu et al., 2022). According to Katzman and colleagues, 2003, the application of methyl nicotinate to the skin induces local hyperemia by triggering the release of prostaglandins D_2_ and E_2_, which causes vasodilation in the peripheral dermal capillaries. This release of prostaglandins may also be linked to the skin’s exposure to UV radiation, a stressor that generates ROS (Sauce et al., 2021).

Table 4 presents the values of the AUC and angular coefficient, as ratios between the parameters obtained in each treated area and those obtained in the untreated ones. Polyphenols have the potential to modulate pro-inflammatory cytokines, such as IL-6, inhibiting their release and preventing exacerbated inflammatory processes (Abad et al., 2011). It is known that UVB rays induce the production of several cytokines. For instance, Yoshizumi and colleagues, 2008, when exposed human keratinocytes (HaCaT cells) in culture to UVB radiation, found ILs (interleukins), like, 1β, 6, and 8; TNF-α (tumor necrosis factor); IFN-γ (interferon), MIP-1β (macrophage inflammatory protein; and G-CSF (granulocyte-colony stimulating factor) (Scwarz and Luger, 1989). Among the compounds mentioned in the literature that help prevent inflammation from intensifying are naringenin and kaempferol (Shin et al., 2017), supporting the results obtained in this investigation regarding the AUC for both samples and the angular coefficient during the hyperemia phase for naringenin.

**TABLE 4:** Ratios of the area under the curve (AUC) and angular coefficient during the hyperemia phase of the investigated samples.

Parameter	Sample	Ratio (mean ± standard deviation)	*p*-value
Area under the curve	Chlorogenic acid	0.855 ± 0.572	0.1189
	Apigenin	0.811 ± 0.305	0.3910
	Kaempferol	0.694 ± 0.337	0.0353 *
	Naringenin	0.559 ± 0.290	0.0085 *
	Blank gel	0.853 ± 0.357	0.2820
Angular coefficient	Chlorogenic acid	0.357 ± 0.242	0.6257
	Apigenin	0.264 ± 0.148	0.0906
	Kaempferol	0.260 ± 0.189	0.0785
	Naringenin	0.222 ± 0.178	0.0031 *
	Blank gel	0.686 ± 0.250	0.3808

Chlorogenic acid and apigenin did not show significantly different responses from the vehicle without active ingredient for AUC and angular coefficient (*p* > 0.05). Kaempferol did not show a significantly different result from the blank gel for the angular coefficient, however, this compound had a significant action in diminishing the AUC, indicating some level of ability to reduce the intensity of the inflammatory response compared to the blank gel. Our results contrasted with Shin and colleagues, 2017, who observed a significant anti-inflammatory activity of apigenin in an animal model (mice) due to the inhibition of prostaglandin E_2_ production (Shin et al., 2017). The results for apigenin in terms of AUC and angular coefficient, particularly in this investigation involving subjects, may be explained by some protocol conditions, like the concentration of the active substance which was insufficient to show explicit efficacy, another type of vehicle could have been tested (an emulsion, for instance), and the participants’ treatment could have been prolonged, instead of a single application. It is worth noting that apigenin, chlorogenic acid, and kaempferol also did not yield significant results in protecting the SC against lipid peroxidation through the HPLC-TBARS-EVSC protocol. Reinforcing, differences in the AUC between the naringenin and the control were statistically significant (*p* < 0.05), as were differences in the angular coefficient during the hyperemia phase (*p* < 0.05). This response profile is consistent with findings by Frade, 2020, who observed that inducing inflammation in RAW 264.7 macrophages and subsequently treating them with naringenin (50 and 100 nM) reduced the secretion of IL-6 and TNF. This suggests that phenolic compounds may exert anti-inflammatory effects by inhibiting cytokine release.

**FIGURE 5:**
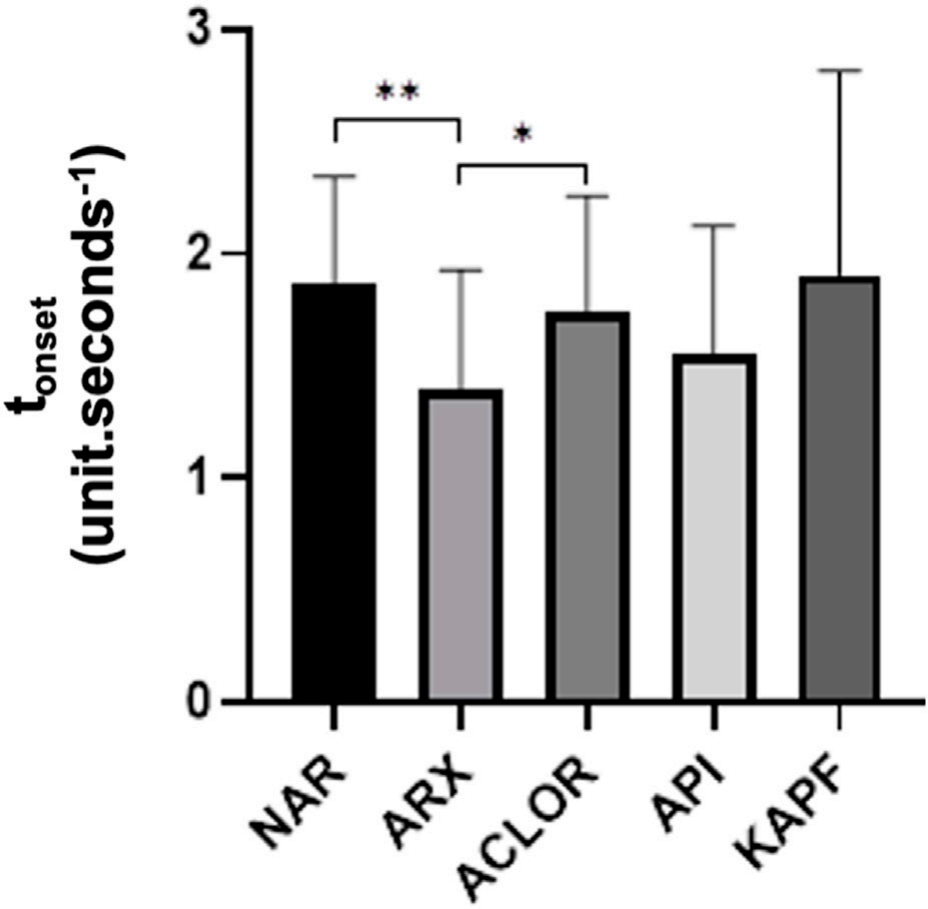
Time to increase perfusion units (t_onset_) of the samples as the ratio of chlorogenic acid (ACLOR), apigenin (API), blank gel (ARX), kaempferol (KAPF), and naringenin (NAR). Legend: **p = 0.005 and *p = 0.0168.

Regarding the t_onset_ (Figure 5), both chlorogenic acid and naringenin exhibited a significant response in delaying this parameter (*p* < 0.05). However, only naringenin demonstrated the ability to reduce the intensity of the inflammatory response, as evidenced by the AUC and angular coefficient.

Naringenin (5,7,4′-trihydroxyflavanone) (Figure 1) is a well-known flavonoid largely found in citrus fruits such as lemons, oranges, tangerines, and grapefruits. Its widespread presence in a variety of popular fruits ensures that naringenin is a regular component of the human diet (Tripoli et al., 2007; Amin, et al., 2020). The biological activities of naringenin are diverse and have been the subject of numerous studies (Manchope et al., 2017; Salehi et al., 2019; Stabrauskiene et al., 2022; Cai et al., 2023; Uçar and Göktas, 2023). Among the many effects, one of the most prominent is its potential to act inhibiting the cyclooxygenase-2 (COX-2), an enzyme that plays a crucial role in the inflammation process and is a target of several non-steroidal anti-inflammatory drugs, known as “NSAIDs”. This particular action of naringenin was brought to light in a study by Chao and colleagues in 2010 (Chao et al., 2010) Ref), marking a significant step forward in understanding the molecular basis of its anti-inflammatory properties. Expanding this initial finding, a further publication by Manchope and co-workers in 2016 (Manchope et al., 2016) Ref) provided additional insights into the mechanism by which naringenin exerts its COX-2 regulation. They demonstrated that naringenin was able to inhibit the expression of COX-2 mRNA when induced by superoxide anion (O_2_^−^), a ROS commonly associated with the generation of pain and inflammation by nociception.

In 2020, Elawa and colleagues (Elawa et al., 2020) demonstrated that the effects of the topically applied methyl nicotinate (MN) are primarily mediated by the prostaglandin pathway. Pre- and post-treatment with NSAIDs notably reduced the vasodilatory response after MN application, suggesting that the prostaglandin pathway plays an important role in the mechanism of action of the topically applied MN. The aforementioned literature is in agreement with our results obtained in the assays with MN, therefore, corroborating the hypothesis that the superior anti-inflammatory effect of naringenin observed here may be, at least partially, attributed to COX-2 inhibition. Given that other targets and biochemical pathways, besides prostaglandin pathway modulation, could also be potentially affected by naringenin, our future efforts will aim for a comprehensive investigation of the molecular basis of the naringenin’s topical effect toward better understanding its anti-inflammatory mechanism.

Despite the innovation of using one *ex vivo* (HPLC-TBARS-EVSC) protocol and one *in vivo* test (laser Doppler flowmetry) to challenge the efficacy of four polyphenols topically applied, being both assays considered non-invasive, it is noteworthy to mention that the investigation had a limitation the number of participants, however, the results were discriminatory according to the statistical treatment. Also, for future investigations aiming at improving this protocol, the inclusion-exclusion criteria may be added to the consumption or not of food supplements that may interfere with the cutaneous tissue behavior. We may highlight that a strength of our research was the experimental design that had not been previously explored for the investigation of the topical anti-inflammatory performance of chlorogenic acid, apigenin, kaempferol, and naringenin in humans. Considering that exposure to UV radiation can promote an inflammatory response, a strategy to prevent damage caused by sun exposure would be the use of active ingredients that inhibit the inflammatory response (Zhu et al., 2022). In light of the results obtained by the applied protocols, naringenin, in addition to protecting the SC from lipid peroxidation, was able to reduce the effect of methyl nicotinate stress on the participants.

## 4 Conclusion

Through the utilization of the HET-CAM assay for *in vitro* safety assessment, chlorogenic acid, apigenin, kaempferol, and naringenin were classified as non-irritating active ingredients. This classification was based on their lack of adverse reactions within the vascularization of the chorioallantoic membrane. To assess the protective capabilities of chlorogenic acid, apigenin, kaempferol, and naringenin against lipid peroxidation in the SC, a comprehensive analysis was conducted using the HPLC-TBARS-EVSC protocol. It was observed that only naringenin exhibited a significant reduction in epidermal lipoperoxidation, indicating superior anti-radical potential. Conversely, chlorogenic acid, apigenin, and kaempferol displayed a pro-oxidant profile under the specified test conditions. The laser Doppler flowmetry suggested the anti-inflammatory potential of naringenin, kaempferol, and chlorogenic acid, with naringenin showing superior efficacy involving all parameters quantified (area under the curve, angular coefficient, and t_onset_). Naringenin emerged as the only polyphenol capable of reducing the intensity of the inflammatory response induced by methyl nicotinate solution in the participants, compared to the gel lacking an active ingredient and the untreated area.

## Data Availability

The raw data supporting the conclusion of this article will be made available by the authors, without undue reservation.

## References

[B1] Abad M. J. Bedoya L. M. Apaza L. Bermejo P. (2011). Anti-infective flavonoids: an overview in bioactive natural products: opportunities and challenges in medicinal chemistry. Singapura World Sci., 443–474. 10.1142/9789814335386_0009

[B2] ALLTOX. Non-animal Methods for Toxicity Testing (2016) Table of validated and accepted alternative methods. Validation and regulatory acceptance status of alternative test methods and testing strategies. Available at: http://www.alltox.org/ttrc/validation-ra/validated-ra-methods.html (Acessed October 23, 2021).

[B3] Alonso C. Barba C. Rubio L. Scott S. Kilimnik A. Coderch L. (2009). An *ex vivo* methodology to assess the lipid peroxidation in stratum corneum. J. Photochem. Photobiol. Biol. 97, 71–76. 10.1016/j.jphotobiol.2009.08.003 19747839

[B4] Amin I. Majid S. Farooq A. Wani H. Noor F. Khan R. (2020). Naringenin (4,5,7-trihydroxyflavanone) as a potent neuroprotective agent: from chemistry to medicine. Nat. Prod. Chem. 65, 271–300. 10.1016/B978-0-12-817905-5.00008-1

[B5] Ariede M. B. Morocho-Jácome A. L. Candido T. M. Lourenço F. R. Kato E. T. M. Lima F. V. (2020). Is the *Botryococcus braunii* dry biomass an adjuvant for Anti-UVB topical formulations? Sci. Pharm. 88, 22–29. 10.3390/scipharm88020022

[B6] Bastos A. S. Loureiro A. P. M. Oliveira T. F. Corbi S. C. Caminaga R. M. S. Rossa J. C. (2012). Quantitation of malondialdehyde in gingival crevicular fluid by a high-performance liquid chromatography-based method. Anal. Biochem. 423 (1), 141–146. 10.1016/j.ab.2012.01.016 22330745

[B7] Bedard K. Krause K. H. (2007). The NOX family of ROS-generating NADPH oxidases: physiology and pathophysiology. Physiol. Rev. 87 (1), 245–313. 10.1152/physrev.00044.2005 17237347

[B8] Cai J. Wen H. Zhou H. Zhang D. Lan D. Liu S. (2023). Naringenin: a flavanone with anti-inflammatory and anti-infective properties. Biomed. Pharmacother. 164, 114990. 10.1016/j.biopha.2023.114990 37315435

[B9] Cândido T. M. Ariede M. B. Pinto C. A. S. Lima F. V. Magalhães W. V. Pedro N. M. E. (2022). Rosmarinic acid multifunctional sunscreen: comet assay and *in vivo* establishment of cutaneous attributes. Cosmetics 9, 141. 10.3390/cosmetics9060141

[B10] Chao C. L. Weng C. S. Chang N. C. Lin J. S. Kao S. T. Ho F. M. (2010). Naringenin more effectively inhibits inducible nitric oxide synthase and cyclooxygenase-2 expression in macrophages than in microglia. Nutr. Res. 30 (12), 858–864. 10.1016/j.nutres.2010.10.011 21147369

[B11] Del Rio D. Stewart A. J. Pellegrini N. (2005). A review of recent studies on malondialdehyde as toxic molecule and biological marker of oxidative stress. Nutr. Metab. Cardiovasc. Dis. 15, 316–328. 10.1016/j.numecd.2005.05.003 16054557

[B12] Elawa S. Mirdell R. Farnebo S. Tesselaar E. (2020). Skin blood flow response to topically applied methyl nicotinate: possible mechanisms. Skin. Res. Technol. 26 (3), 343–348. 10.1111/srt.12807 31777124

[B13] Frade A. C. M. (2020) Fitoquímica e avaliação do efeito sobre a liberação de citocinas pró-inflamatórias de espécies do gênero baccharis. Belo Horizonte (MG): University of Minas Gerais. dissertation/master’s thesis.

[B14] Frantzen M. Regoli F. Ambrose W. Nahrgang J. Geraudie P. Benedetti M. (2016). Biological effects of mechanically and chemically dispersed oil on the Icelandic scallop (Chlamys islandica). Ecotoxicol. Environ. Saf. 127, 95–107. 10.1016/j.ecoenv.2016.01.011 26809079

[B15] Instituto Nacional de Câncer (Brasil) (2022) Câncer. Tipos de câncer. Câncer de pele melanoma. Rio de Janeiro: INCA. Available at: https://www.gov.br/inca/pt-br/assuntos/cancer/tipos/pele-melanoma (Accessed august, 2022).

[B16] International Agency for Research on Cancer (IARC) (2012) A review of human carcinogens. Radiation. IARC monographs on the evaluation of carcinogenic risks to humans, volume D. Lyon, France: IARC.

[B17] Jansen R. Wang S. Burnett M. Osterwalder U. Lim H. W. (2013). Photoprotection. Part I. Photoprotection by naturally occurring physical, and systemic agentes. J. Am. Acad. Dermatol 69 (6), 853. 10.1016/j.jaad.2013.08.021 24238179

[B18] Junqueira L. C. Carneiro J. (2013) Histologia básica. 12. Rio de Janeiro: Guanabara Koogan.

[B19] Kalweit S. Besoke R. Spielmann G. H. (1990). A national validation project of alternative methods to the Draize rabbit eye. Toxicol. Vitro 4/5, 702–706. 10.1016/0887-2333(90)90147-l 20702261

[B20] Kim K. C. Piao M. J. Cho S. J. Lee N. H. Hyun J. W. (2012). Phloroglucinol protects human keratinocytes from ultraviolet B radiation by attenuating oxidative stress. Photodermatol. Photoimmunol. Photomed. 28, 322–331. 10.1111/phpp.12010 23126295

[B21] Kolarsick P. Kolarsick M. A. Goodwin C. (2011). Anatomy and physiology of the skin. J. Dermatology Nurses' Assoc. 3, 203–213. 10.1097/JDN.0b013e3182274a98

[B22] Lima E. S. Abdalla D. S. P. (2001). Peroxidação lipídica: mecanismos e avaliação em amostras biológicas. Rev. Bras. Ciências Farm. 37, 293–303.

[B23] Manchope M. Calixto-Campos C. Coelho-Silva S. Zarpelon A. Pinho-Ribeiro F. A. Georgetti S. R. (2016). Naringenin inhibits superoxide anion-induced inflammatory pain: role of oxidative stress, cytokines, nrf-2 and the NO−cGMP−PKG−KATPChannel signaling pathway. PlosOne 5, e0153015–e0153020. 10.1371/journal.pone.0153015 PMC4821586 27045367

[B24] Manchope M. F. Casagrande R. Verri W. A. (2017). Naringenin: an analgesic and anti-inflammatory citrus flavanone. Oncotarget 8, 3766–3767. 10.18632/oncotarget.14084 28030851 PMC5354790

[B25] Marques G. Hiraishi C. F. Macedo P. I. S. Pinto C. A. S. O. Gregório J. Rosado C. (2023). HPLC-TBARS-EVSC (high-performance liquid chromatography-thiobarbituric acid reactive substances-*ex vivo* stratum corneum) protocol: selection of the subjects and approach to present the results. Int. J. Cosmet. Sci. 45, 647–654. 10.1111/ics.12874 37265451

[B26] Martinez R. Felipe A Pinho-Ribeiro F. Steffen V. Caviglione C. V. Vignoli J. A. Barbosa D. S. (2016). Naringenin inhibits UVB irradiation-induced inflammation and oxidative stress in the skin of hairless mice. J. Nat. Prod. 78, 1647–1655. 10.1021/acs.jnatprod.5b00198 26154512

[B27] Musakhanian J. Rodier J.-D. Dave M. (2022). Oxidative stability in lipid formulations: a review of the mechanisms, drivers, and inhibitors of oxidation. AAPS Pharm. Sci. Tech. 23, 151. 10.1208/s12249-022-02282-0 35596043

[B28] National Institute of Enviromental Health Sciences (NIEHS) (2021) ICCVAM test method evaluation report: *in vitro* ocular toxicity test methods for identifying severe irritants and corrosives. Durham, NC: NHI Publication No: 07-4517. Available at: http://ntp.niehs.nih.gov/iccvam/docs/ocutox_docs/oteval/otevalrpt.pdf (Accessed October, 2021).

[B29] Pescia A. C. Astolfi P. Puglia C. Bonina F. Perrotta R. Herzog B. (2012). On the assessment of photostability of sunscreens exposed to UVA irradiation: from glass plates to pig/human skin, which is best? Int. J. Pharm. 427, 217–223. 10.1016/j.ijpharm.2012.02.001 22343131

[B30] Petersen L. (2013). Direct comparison of laser Doppler flowmetry and laser Doppler imaging for assessment of experimentally-induced inflammation in human skin. Inflamm. Res. 62, 1073–1078. 10.1007/s00011-013-0668-2 24114290

[B31] Pilz J. Meineke I. Gleiter C. (2000). Measurement of free and bound malondialdehyde in plasma by highperformance liquid chromatography as the 2,4dinitrophenylhydrazine derivative. J. Chromatogr. B Anal. Technol. Biomed. Life Sci. 742, 315–325. 10.1016/s0378-4347(00)00174-2 10901136

[B32] Reinehr C. P. H. Bakis R. M. (2019). Actinic keratoses: review of clinical, dermoscopic, and therapeutic aspects. An. Bras. Dermatol. 94, 637–657. 10.1016/j.abd.2019.10.004 31789244 PMC6939186

[B33] Ruscinc N. Morocho-Jácome A. L. Martinez R. M. Magalhaes W. V. Escudeiro C. C. Giarolla J. (2022). *Vaccinium myrtillus* L. extract associated with octocrylene, bisoctrizole, and titanium dioxide: *in vitro* and *in vivo* tests to evaluate safety and efficacy. J. Cosmet. Dermatology 21, 4765–4774. 10.1111/jocd.14779 35029052

[B34] Salehi B. Fokou P. Sharifi-Rad M. Zucca P. Pezzani R. Martins N. (2019). The therapeutic potential of naringenin: a review of clinical trials. Pharmaceuticals 12, 11–18. 10.3390/ph12010011 30634637 PMC6469163

[B35] Sarnik I. H. Sochor O. (2007). Laser Doppler fluxmetry. Biomed. Pap. Med. Fac. Univ. Palacky. Olomouc. Czech. Repub. 151, 143–146. 10.5507/bp.2007.028 17690759

[B36] Sauce R. Pinto C. A. S. O. Ayala-Jara C. Prieto Z. A. Velasco M. V. R. Baby A. R. (2021). Preliminary protocol development of a HPLC-TBARS-EVSC (*ex vivo* stratum corneum) assay for skin research: application in a sunscreen system. Sci. Pharm. 89, 17. 10.3390/scipharm89020017

[B37] Schwarz T. Luger T. A. (1989). New trends in photobiology. J. Photochem. Photobiol. B, Biol. 4 (1), 1–13. 10.1016/1011-1344(89)80097-1 2509656

[B38] Shin K. C. Hwang I. Choe S. S. Park J. Ji Y. Kim J. I. (2017). Macrophage VLDLR mediates obesity-induced insulin resistance with adipose tissue inflammation. Nat. Commun. 1087, 1087. 10.1038/s41467-017-01232-w PMC5651811 29057873

[B39] Sikurova L. Balis P. Zvarik M. (2011). Penetration of laser light through red blood cell ghosts. J. Photochem. Photobiol. B Biol. 103, 230–233. 10.1016/j.jphotobiol.2011.03.015 21501961

[B40] Šínová R. Pavlík V. Ondrej M. Velebný V. Nešporová K. (2021). Hyaluronan: a key player or just a bystander in skin photoaging? Exp. Dermatol. 31, 442–458. 10.1111/exd.14491 34726319

[B41] Stabrauskiene J. Kopustinskiene D. M. Lazauskas R. Bernatoniene J. (2022). Naringin and naringenin: their mechanisms of action and the potential anticancer activities. Biomedicines 10, 1686. 10.3390/biomedicines10071686 35884991 PMC9313440

[B42] Steiling W. Bracher M. Courtellemont P. Silva O. (1999). The HET-CAM, a useful *in vitro* assay for assessing the eye irritation properties of cosmetic formulations and ingredients. Toxicol. Vitro 13, 375–384. 10.1016/S0887-2333(98)00091-5 20654494

[B43] Sun M. Deng Y. Cao X. Xiao L. Ding Q. Luo F. (2022). Effects of natural polyphenols on skin and hair health: a review. Molecules 27, 7832. 10.3390/molecules27227832 36431932 PMC9695112

[B44] Tobin D. J. (2017). Introduction to skin aging. J. Tissue Viability 26 (1), 37–46. 10.1016/j.jtv.2016.03.002 27020864

[B45] Torres M. Parets S. Fernández-Díaz J. Beteta- Göbel R. Rodríguez-Lorca R. Román R. (2021). Lipids in pathophysiology and development of the membrane lipid therapy: new bioactive lipids. Membranes (Basel) 24, 919. 10.3390/membranes11120919 PMC8708953 34940418

[B46] Tripoli E. La Guardia M. Giammanco S. Di Majo D. Giammanco M. (2007). Citrus flavonoids: molecular structure, biological activity and nutritional properties: a review. Food Chem. 104, 466–479. 10.1016/j.foodchem.2006.11.054

[B47] Uçar K. Göktaş Z. (2023). Biological activities of naringenin: a narrative review based on *in vitro* and *in vivo* studies. Nutr. Res. 119, 43–55. 10.1016/j.nutres.2023.08.006 37738874

[B48] Vellosa J. C. R. Biavatti M. Françóia P. C. O. de Mello B. J. de Almeida A. C. Bueno G. E. (2021). Estresse oxidativo: uma introdução ao estado da arte/Oxidative stress: an introduction to the state of art. Braz. J. Dev. 7 (1), 10152–10168. 10.34117/bjdv7n1-688

[B49] Vertuani S. Ziosi P. Solaroli N. Buzzoni V. Carli M. Lucchi E. (2003). Determination of antioxidant efficacy of cosmetic formulations by non-invasive measurements. Ski. Res. Technol. 9, 245–253. 10.1034/j.1600-0846.2003.00018.x 12877686

[B50] Wang S. Yu H. Wickliffe J. K. (2011). Limitation of the MTT and XTT assays for measuring cell viability due to superoxide formation induced by nano-scale TiO_2_ . Toxicol. Vitro 25, 2147–2151. 10.1016/j.tiv.2011.07.007 21798338

[B51] Yagi K. (1994). Lipid peroxides and related radicals in clinical medicine. Adv. Exp. Med. Biol. 366, 1–15. 10.1007/978-1-4615-1833-4_1 7771246

[B52] Yoshizumi M. Nakamura T. Kato M. Ishioka T. Kozawa K. Wakamatsu K. (2008). Release of cytokines/chemokines and cell death in UVB-irradiated human keratinocytes, HaCaT. Cell Biol. Int. 32 (11), 1405–1411. 10.1016/j.cellbi.2008.08.011 18782623

[B53] Zhao G. Han X. Cheng W. Ni j. Zhang Y. Lin J. (2017). Apigenin inhibits proliferation and invasion, and induces apoptosis and cell cycle arrest in human melanoma cells. Oncol. Rep. 37, 2277–2285. 10.3892/or.2017.5450 28260058

[B54] Zhu Y. Xu W. Yang O. Wang H. Mao W. Zhou H. (2022). Topical application of methyl nicotinate solution enhances peripheral blood collection. Am. Soc. Clin. Pathology 53, 500–503. 10.1093/labmed/lmac033 35639810

